# Supplementary material for: Digital navigation of energy–structure–function maps for hydrogen-bonded porous molecular crystals
Source: Nat Commun. 2021 Feb 5;12:817. doi: 10.1038/s41467-021-21091-w (PMC7865007; doi:10.1038/s41467-021-21091-w)
Supplement: Supplementary file 1 — Supplementary Information [file 41467_2021_21091_MOESM1_ESM.pdf]

Supplementary Information

Digital Navigation of Energy–Structure–Function Maps for Hydrogen-Bonded Porous Molecular Crystals

Chengxi Zhao,[1,2] Linjiang Chen,[*,2,3] Yu Che,[2] Zhongfu Pang,[2] Xiaofeng Wu,[2,3] Yunxiang Lu,[1] Honglai Liu,[1] Graeme M Day,[*,4] and Andrew I. Cooper [*2,3]

1. Key Laboratory for Advanced Materials and School of Chemistry and Molecular Engineering, East China University of Science and Technology, Shanghai, China.
2. Leverhulme Research Centre for Functional Materials Design, Materials Innovation Factory and Department of Chemistry, University of Liverpool, Liverpool, UK.
3. Key Laboratory for Advanced Materials and Joint International Research Laboratory of Precision Chemistry and Molecular Engineering, Feringa Nobel Prize Scientist Joint Research Centre, School of Chemistry and Molecular Engineering, East China University of Science and Technology, Shanghai, China.
4. Computational Systems Chemistry, School of Chemistry, University of Southampton, Southampton, UK.

Table of Contents

1 Supplementary Methods 2

2 Supplementary Figures 4

1 Supplementary Methods

A total of 18 pore descriptors were used to describe the porosity space of the predicted crystal structures:

1, crystal density (g cm^{-3});

2–4, pore diameters (Å): the largest included sphere (D_i), the largest free sphere (D_f) and the largest included sphere along the free sphere path (D_{if});

5–8, accessible surface area (m^2 g^{-1}), non-accessible surface area (m^2 g^{-1}), accessible volume (cm^3 g^{-1}), non-accessible volume (cm^3 g^{-1});

9–18, variants based on the surface areas and pore volumes of individual channels (accessible) and pockets (non-accessible) to capture, to some extent, the heterogeneity of the pore space within a crystal structure:

- average of the accessible surface areas divided by the corresponding accessible volumes for all individual channels

$$A_{acc} = \frac{1}{n}\sum_{n=1}^{n}\frac{S_{n,acc}}{V_{n,acc}}$$

- median of the accessible surface areas divided by the corresponding accessible volumes for all individual channels

$$M_{acc} = Median(\frac{S_{1,acc}}{V_{1,acc}}, \frac{S_{2,acc}}{V_{2,acc}} \cdots \frac{S_{n,acc}}{V_{n,acc}})$$

- variance of the accessible surface areas divided by the corresponding accessible volumes for all individual channels

$$\sigma^2{}_{acc} = \frac{1}{n}\sum_{n=1}^{n}(\frac{S_{n,acc}}{V_{n,acc}} - A_{acc})^2$$

- maximum of the accessible surface areas divided by the corresponding accessible volumes for all individual channels

$$P_{max,acc} = Max(\frac{S_{1,acc}}{V_{1,acc}}, \frac{S_{2,acc}}{V_{2,acc}} \cdots \frac{S_{n,acc}}{V_{n,acc}})$$

- Minimum of the accessible surface areas divided by the corresponding accessible volumes for all individual channels

$$P_{min,acc} = Min(\frac{S_{1,acc}}{V_{1,acc}}, \frac{S_{2,acc}}{V_{2,acc}} \cdots \frac{S_{n,acc}}{V_{n,acc}})$$

- average of the non-accessible surface areas divided by the corresponding non-accessible volumes for all individual pockets

$$A_{nacc} = \frac{1}{n}\sum_{n=1}^{n}\frac{S_{n,nacc}}{V_{n,nacc}}$$

- median of the non-accessible surface areas divided by the corresponding non-accessible volumes for all individual pockets

$$M_{nacc} = Median(\frac{S_{1,nacc}}{V_{1,nacc}}, \frac{S_{2,nacc}}{V_{2,nacc}} \cdots \frac{S_{n,nacc}}{V_{n,nacc}})$$

- variance of the non-accessible surface areas divided by the corresponding non-accessible volumes for all individual pockets

$$\sigma^2{}_{nacc} = \frac{1}{n}\sum_{n=1}^{n}(\frac{S_{n,nacc}}{V_{n,nacc}} - A_{nacc})^2$$

- maximum of the non-accessible surface areas divided by the corresponding non-accessible volumes for all individual pockets

$$P_{max,nacc} = Max(\frac{S_{1,nacc}}{V_{1,nacc}}, \frac{S_{2,nacc}}{V_{2,nacc}} \cdots \frac{S_{n,nacc}}{V_{n,nacc}})$$

- Minimum of the non-accessible surface areas divided by the corresponding non-accessible volumes for all individual pockets

$$P_{min,nacc} = Min(\frac{S_{1,nacc}}{V_{1,nacc}}, \frac{S_{2,nacc}}{V_{2,nacc}} \cdots \frac{S_{n,nacc}}{V_{n,nacc}})$$

where n is the number of channels or pockets, $S_{n,acc}$ and $V_{n,acc}$ are the accessible surface area and accessible volume for the nth channel, respectively; $S_{n,nacc}$ and $V_{n,nacc}$ are the non-accessible surface area and non-accessible volume for the nth pocket, respectively.

2 Supplementary Figures

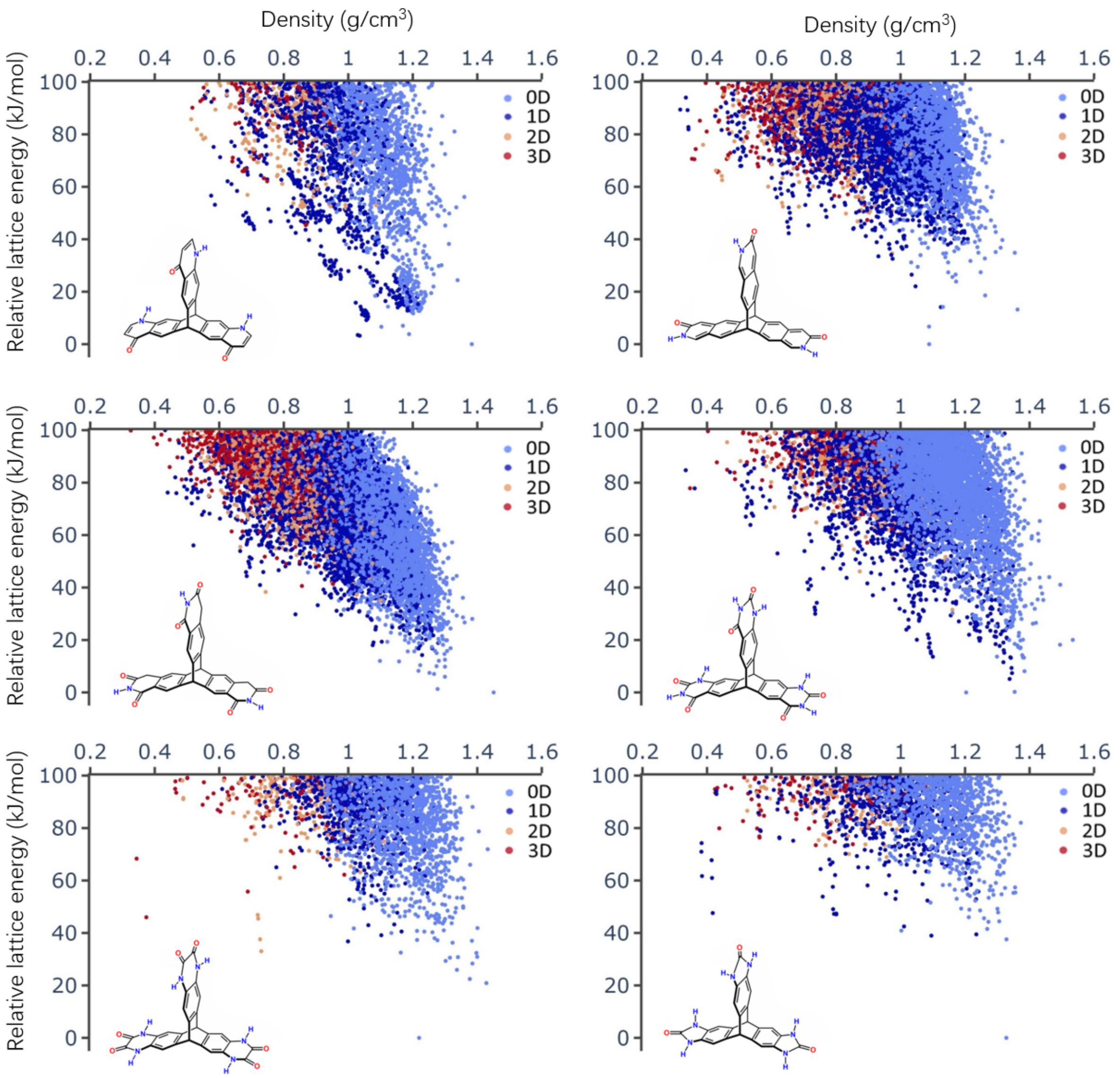

Supplementary Figure 1. Energy–structure–function maps for the molecular building blocks shown in the figure. The data used here are the same as those for Figure 1, but the order of plotting was shuffled with respect to the pore dimensionality; in Figure 1, the 0D data points were first plotted, followed by 1D, 2D and 3D points sequentially.

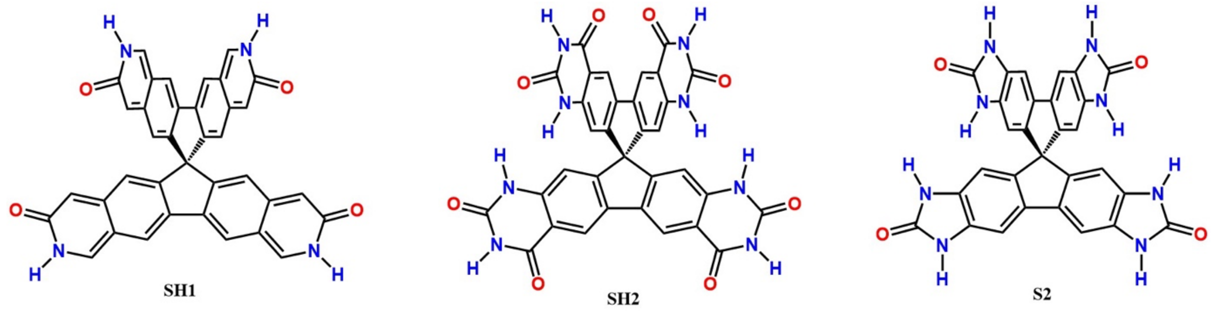

Supplementary Figure 2. Molecular tectons based on a spiro-biphenyl core: **SH1**, **SH2** and **S2**.

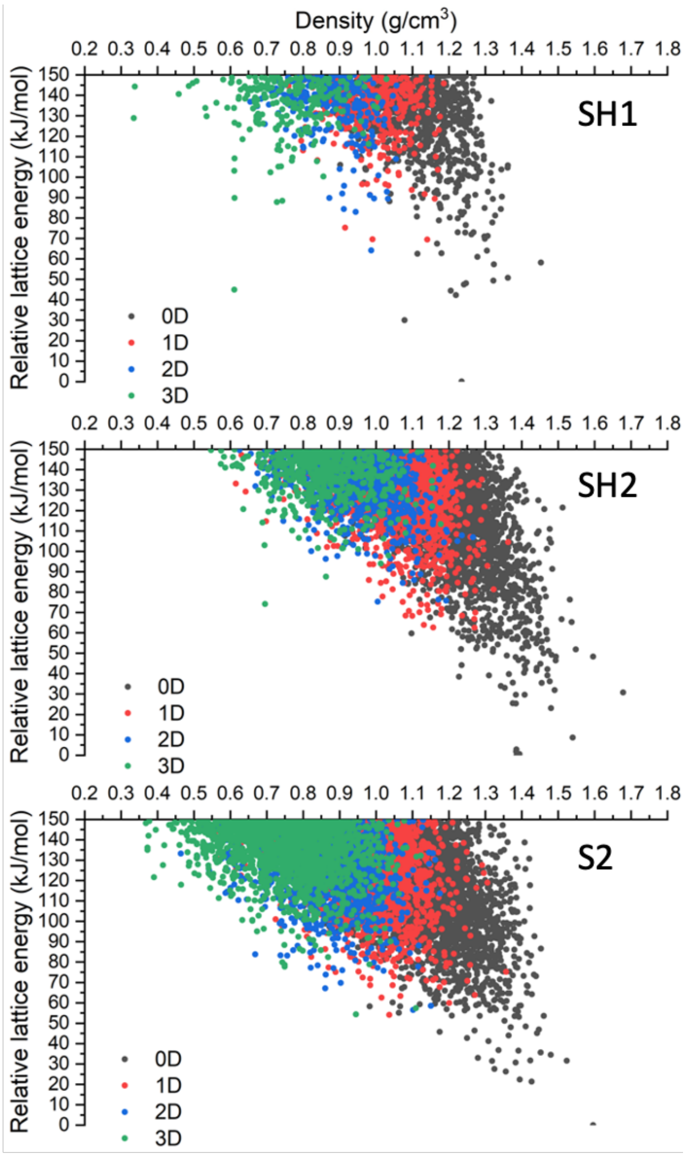

Supplementary Figure 3. CSP energy–density plots for **SH1**, **SH2** and **S2**, with each point corresponding to a computed crystal structure. The symbols are color-coded by the dimensionality of the pore channels, assessed using a probe radius of 1.7 Å.

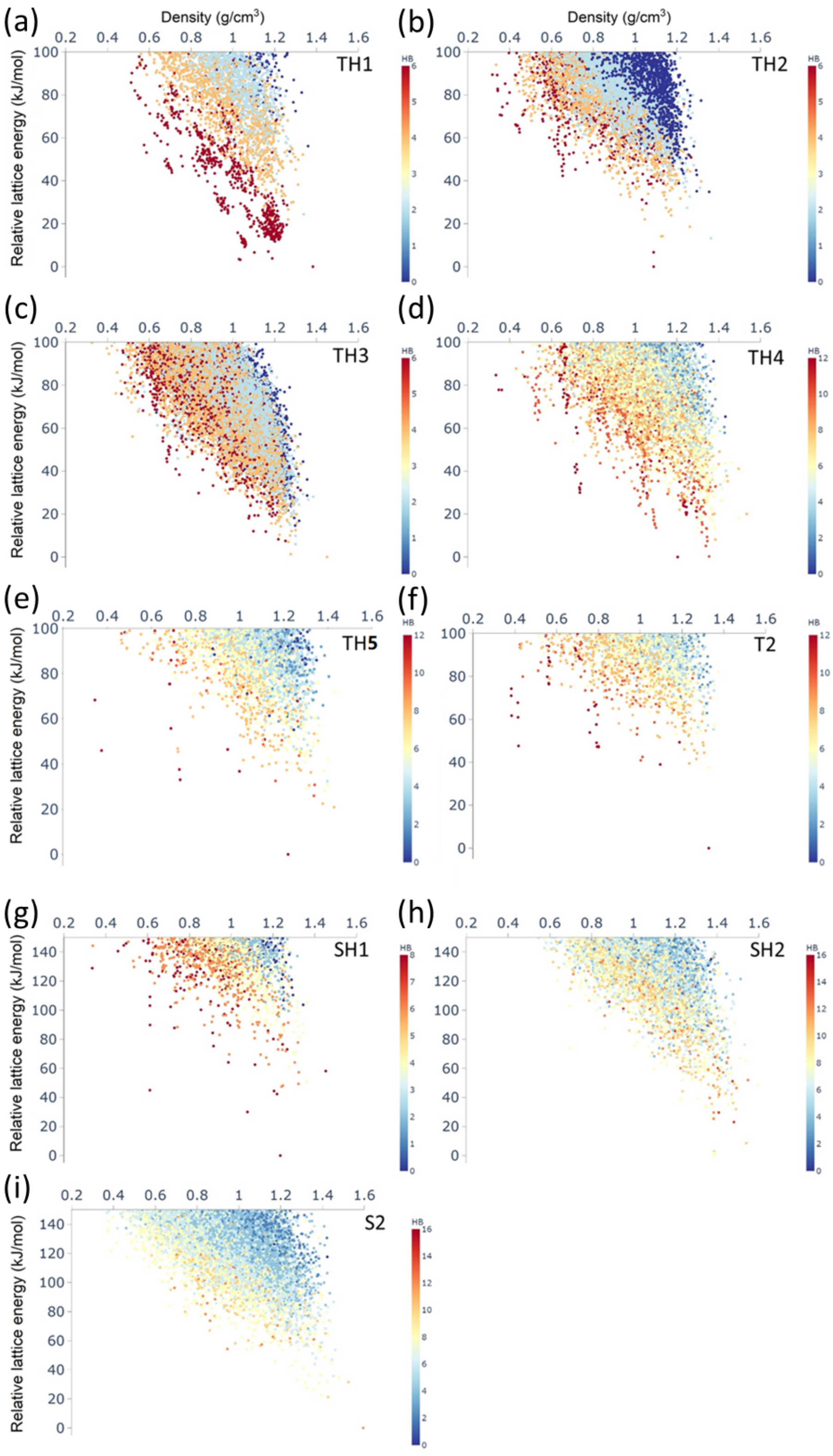

Supplementary Figure 4. **a**–**i**, CSP energy–density landscapes for **TH1** (**a**), **TH2** (**b**), **TH3** (**c**), **TH4** (**d**), **TH5** (**e**), **T2** (**f**), **SH1** (**g**), **SH2** (**h**) and **S2** (**i**), color-coded by the number of intermolecular hydrogen bonds (HB) formed by one molecule with its neighbors in the crystal structure.

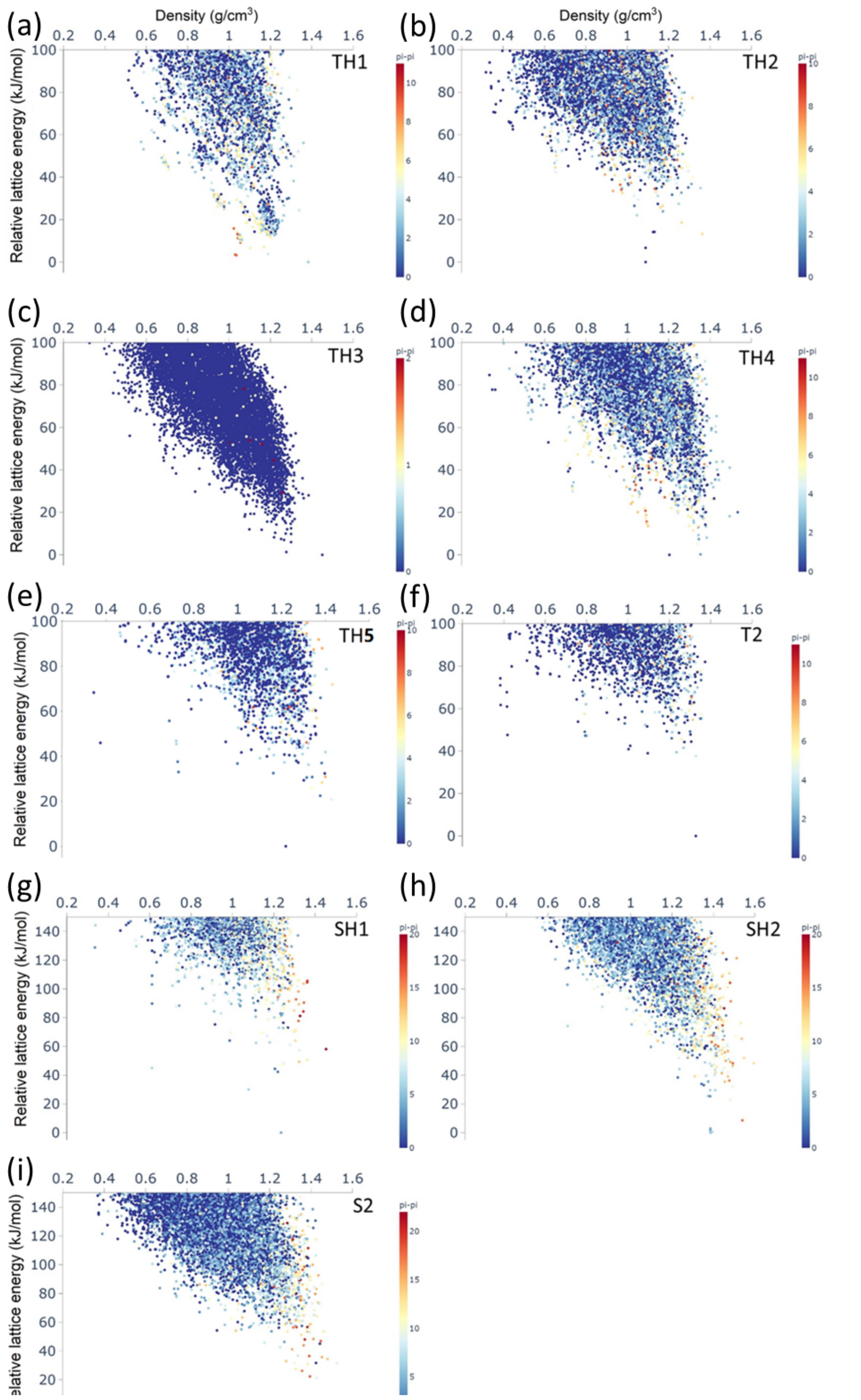

Supplementary Figure 5. a–i, CSP energy–density landscapes for **TH1** (**a**), **TH2** (**b**), **TH3** (**c**), **TH4** (**d**), **TH5** (**e**), **T2** (**f**), **SH1** (**g**), **SH2** (**h**) and **S2** (**i**), color-coded by the number of intermolecular stacking modes (pi–pi) formed by one molecule with its neighbors in the crystal structure.

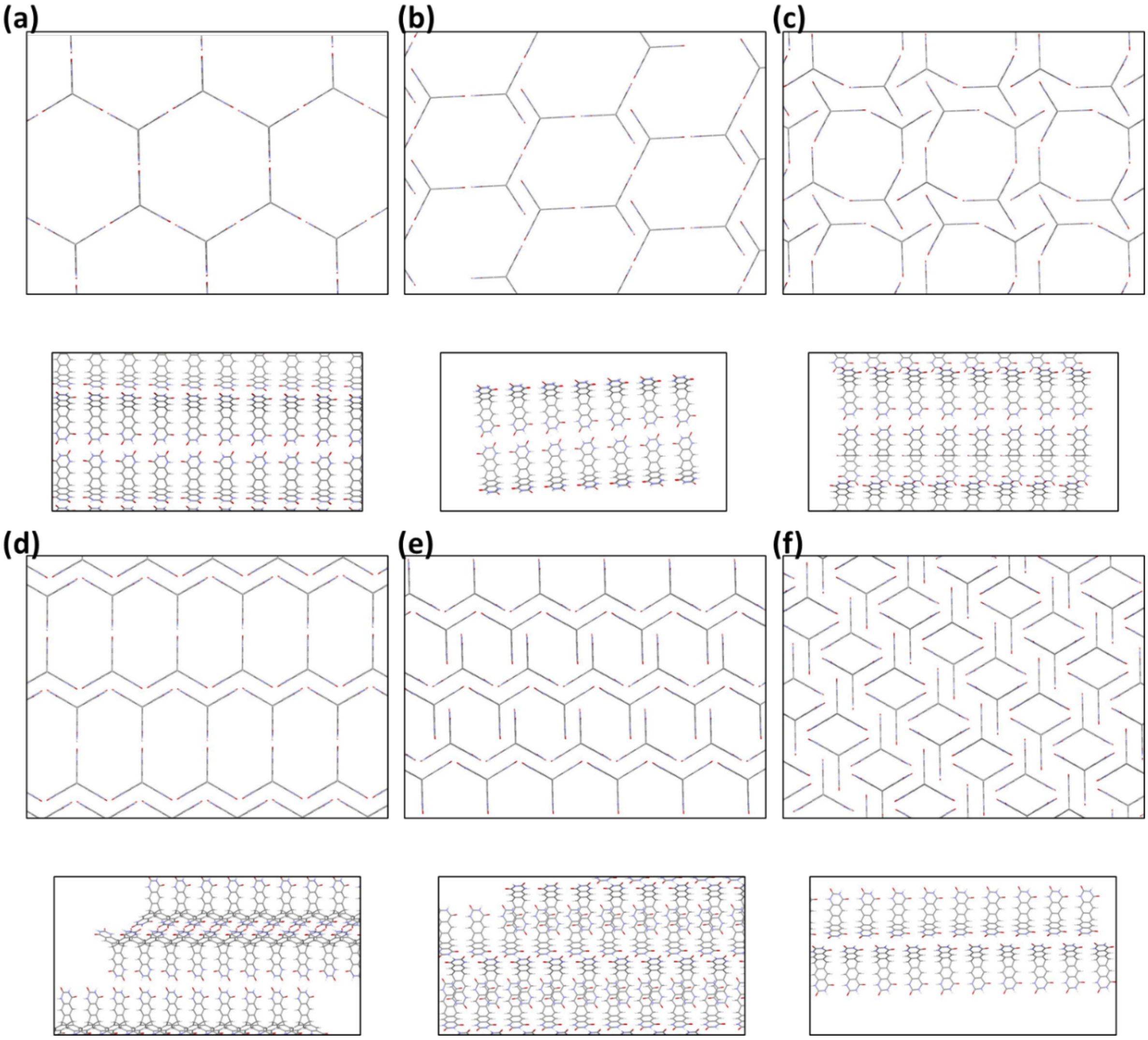

Supplementary Figure 6. TH4 structures highlighted in Figure 4 (main text), viewed along (top) and perpendicular to (bottom) the channel direction: (a) **TH4-A**, (b) **TH4-B,** (c) **TH4-C**, (d) **TH4-D**, (e) **TH4-E**, and (f) **TH4-F**.

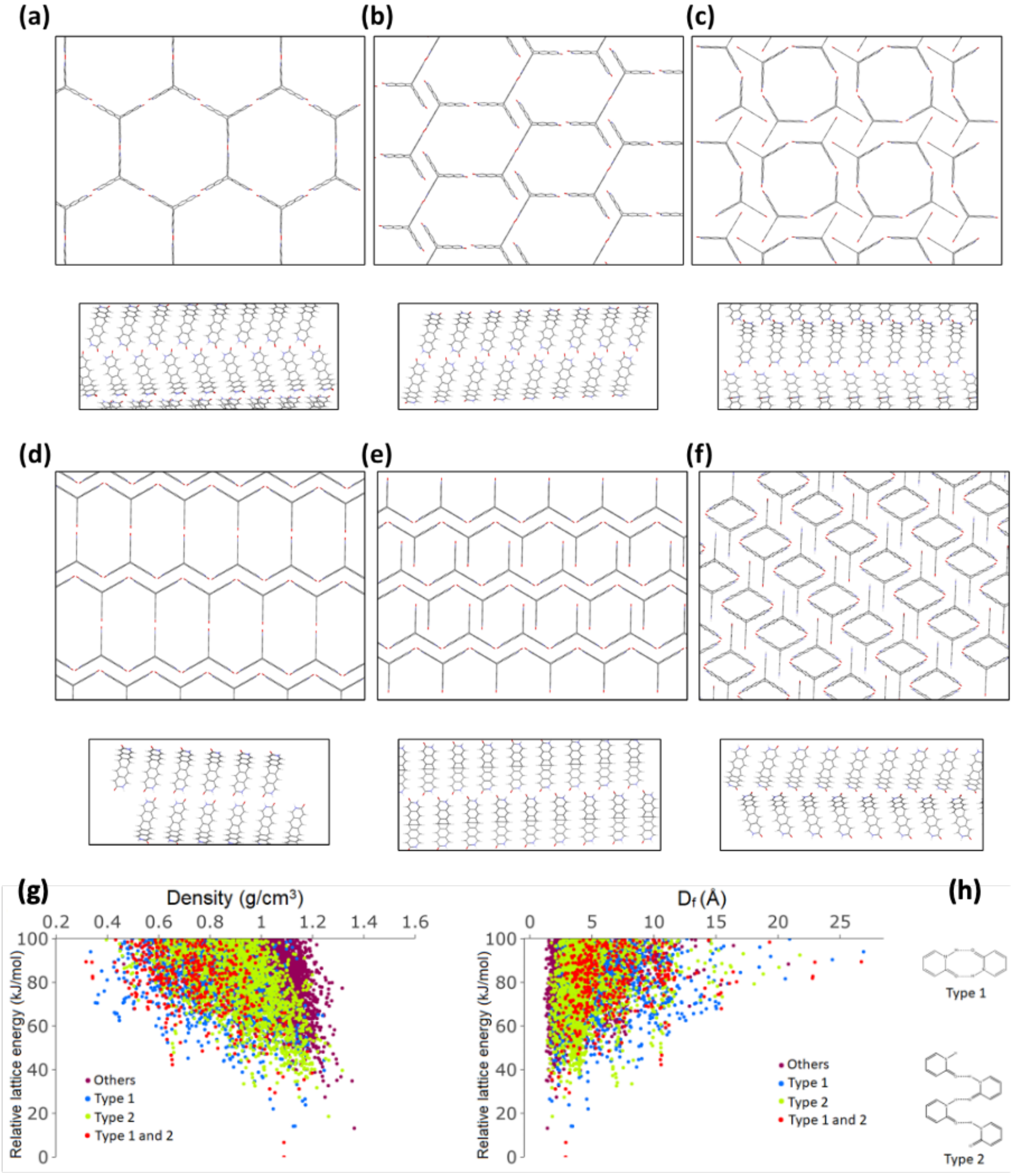

Supplementary Figure 7. TH2 structures highlighted in Figure 4 (main text), viewed along (top) and perpendicular to (bottom) the channel direction: (a) **TH2-A**, (b) **TH2-B,** (c) **TH2-C**, (d) **TH2-D**, (e) **TH2-E**, and (f) **TH2-F**. (g, h) ESF maps (g), color-coded by the types of hydrogen-bonding patterns shown in (h).

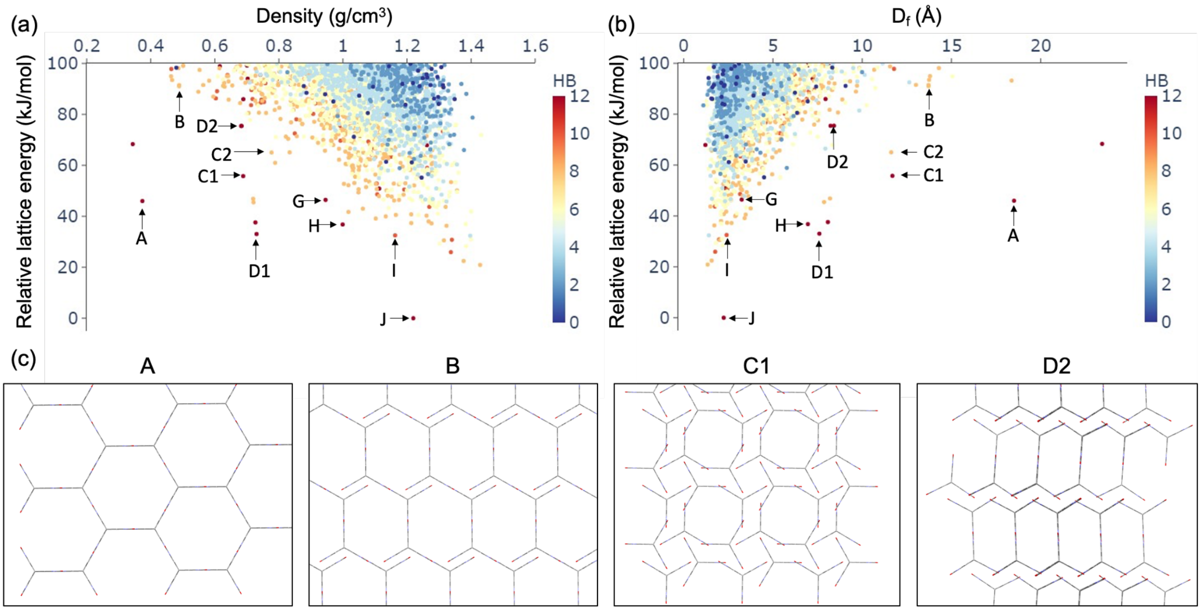

Supplementary Figure 8. ESF maps for **TH5** (a, b), plotted against the crystal density (a) or the largest free sphere diameter (D_f; b); symbols are color-coded by the number of hydrogen bonds (HB) formed by each molecule in the crystal structure. Selected 'landmark' structures A, B, C1 and D2 for **TH5** are displayed in (c) and labelled in (a, b).

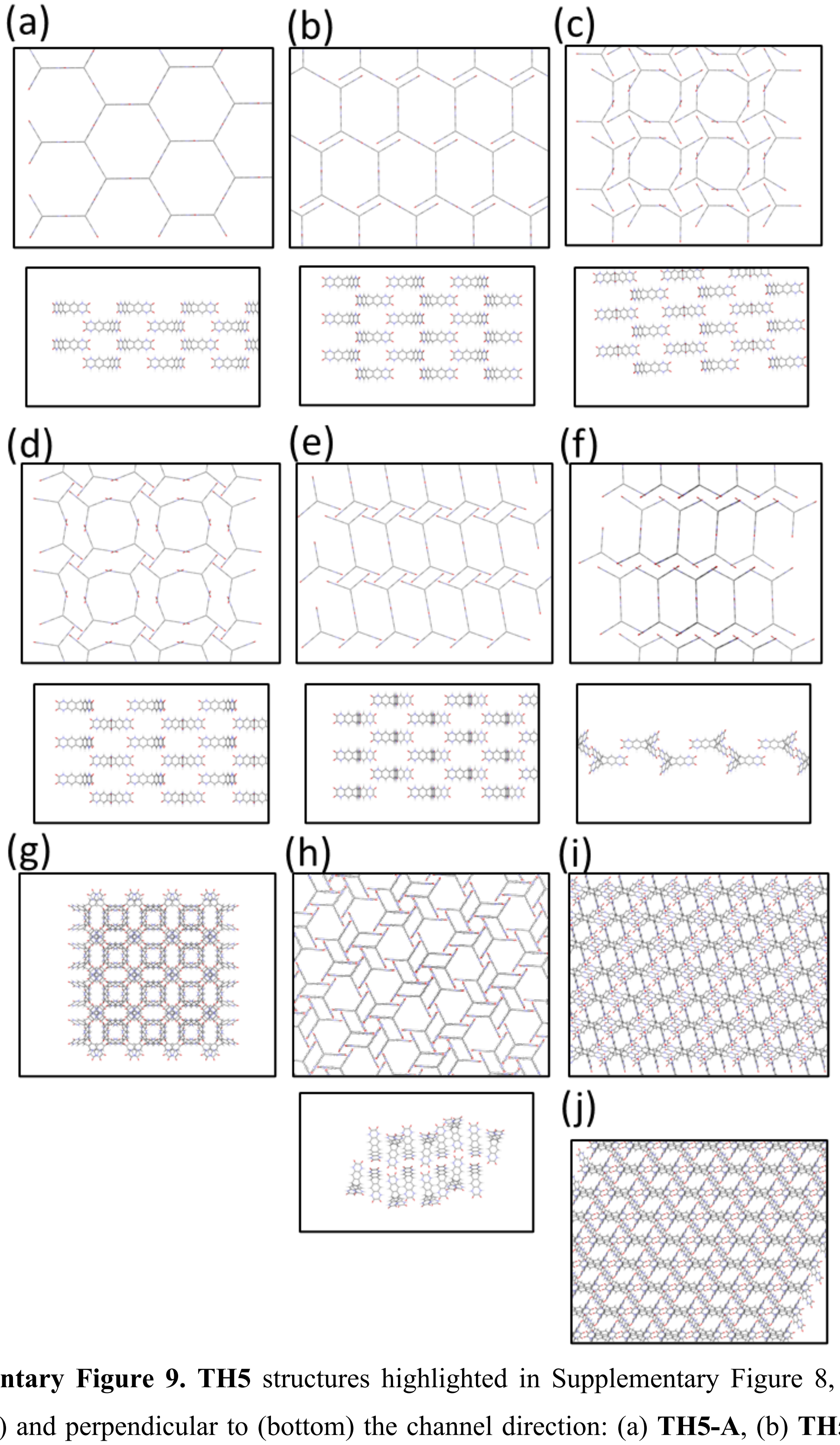

Supplementary Figure 9. TH5 structures highlighted in Supplementary Figure 8, viewed along (top) and perpendicular to (bottom) the channel direction: (a) **TH5-A**, (b) **TH5-B,** (c) **TH5-C1**, (d) **TH5-C2**, (e) **TH5-D1**, (f) **TH5-D2,** (g) **TH5-G**, (h) **TH5-H**, (i) **TH5-I**, and (j) **TH5-J**. Only one view is shown for **TH5-G**, **TH5-I** and **TH5-J** because their pore dimensionality is not 1D.

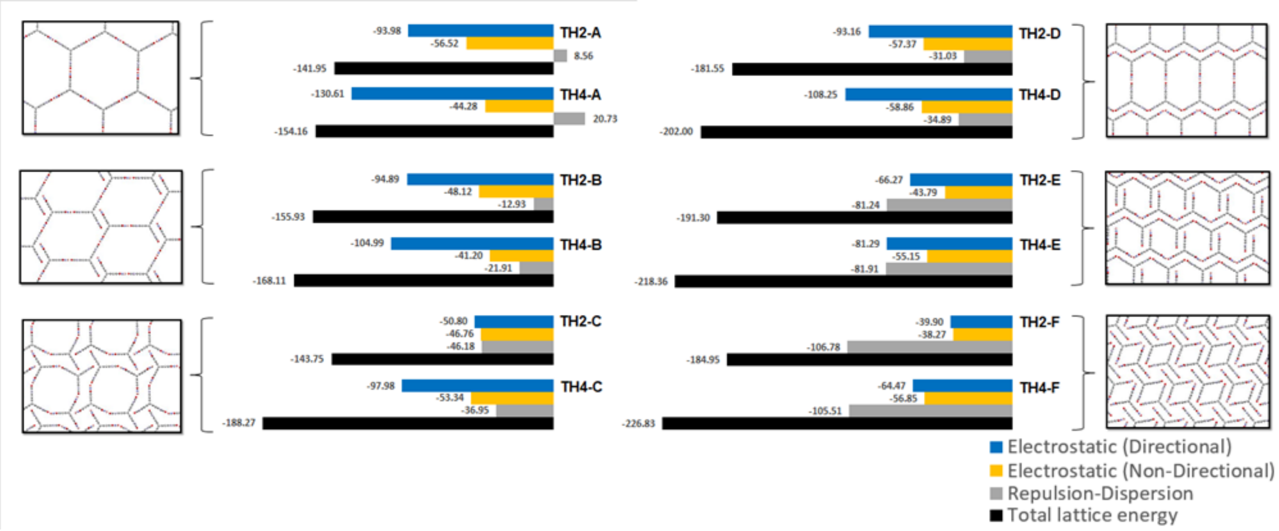

Supplementary Figure 10. Lattice energy decomposition of the landmark structures A–F of **TH2** and **TH4**. The total lattice energy is decomposed into contributions from: (i) intermolecular repulsion-dispersion interaction energy, (ii) intermolecular directional electrostatic interaction energy (charge-dipole and higher order multipole-multipole interactions), and (iii) intermolecular non-directional electrostatic interaction energy (charge-charge interactions only).

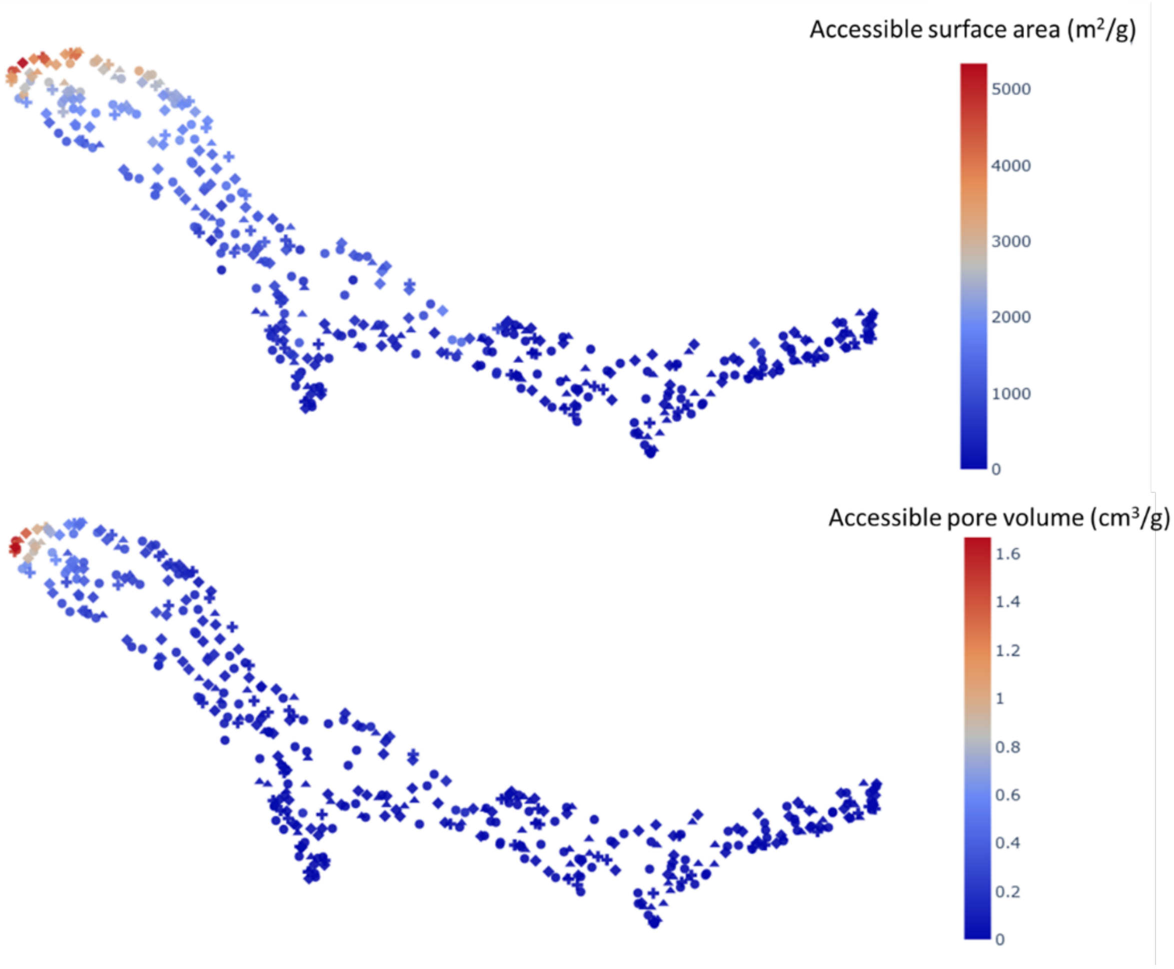

Supplementary Figure 11. 2D UMAP embeddings (the same as the ones shown in Figure 6 of the main text) of the porosity spaces of **TH2** (diamond), **TH4** (circle) **TH5** (triangle) and **T2** (cross), color-coded by the accessible surface area (top) or the accessible pore volume (bottom).

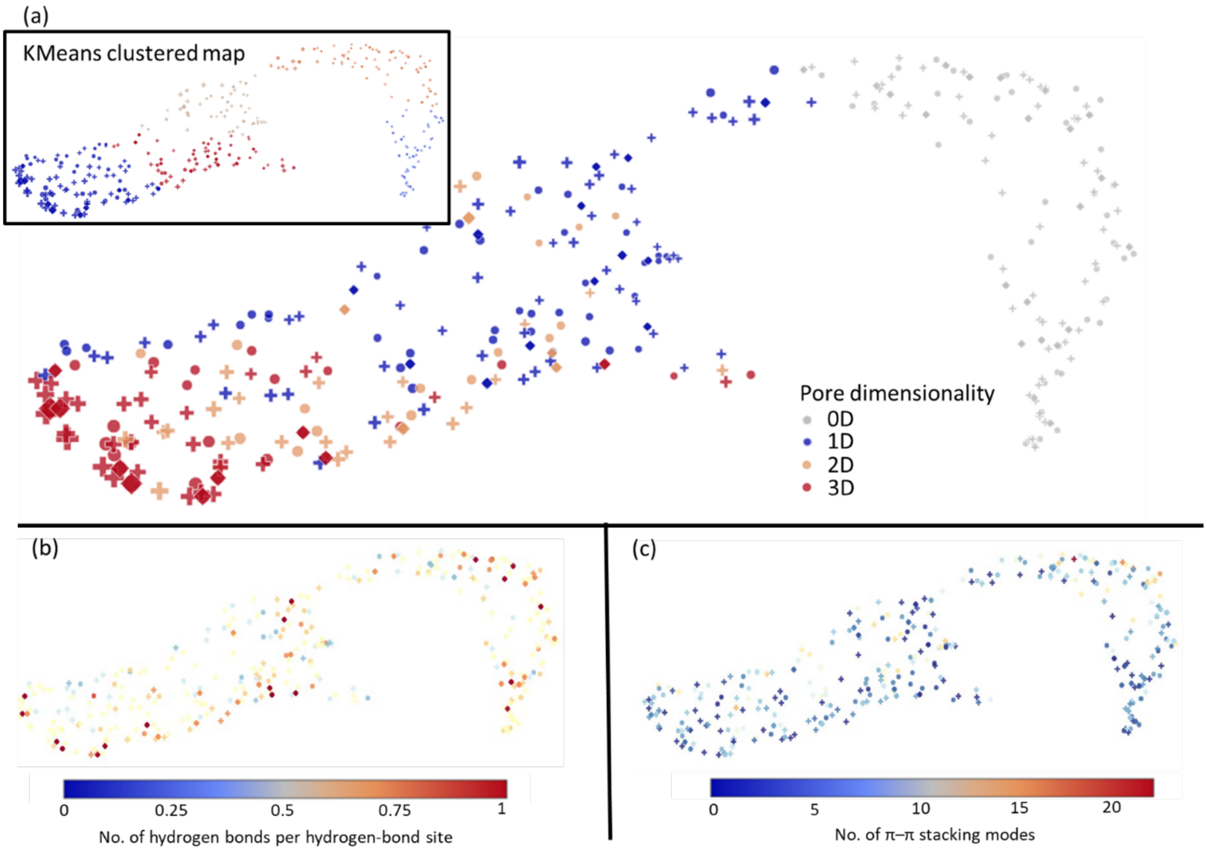

Supplementary Figure 12. 2D UMAP embeddings of the porosity spaces of **SH1** (diamond), **SH2** (circle) and **S2** (cross), color-coded by the pore dimensionality (a), the number (no.) of hydrogen bonds per hydrogen-bond site (b), or the total number of π–π stacking modes of the crystal structure (c); the symbol size is scaled by the accessible surface area.

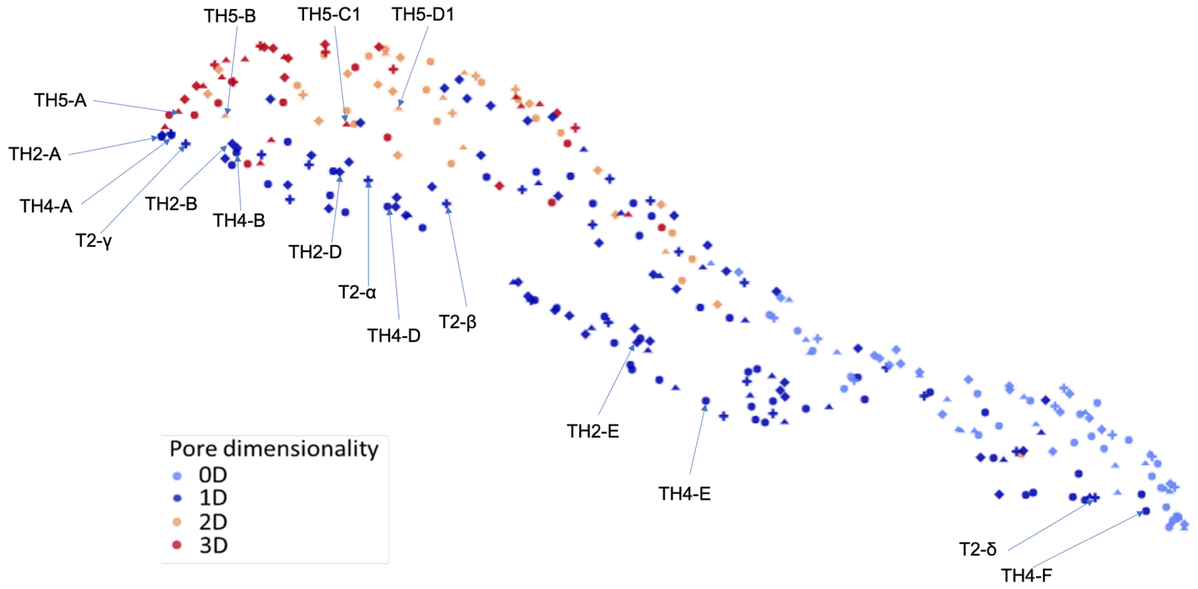

Supplementary Figure 13. 2D UMAP embeddings of the porosity spaces of **TH2** (diamond), **TH4** (circle), **TH5** (triangle) and **T2** (cross), colour-coded by the pore dimensionality. All the points shown are the lowest-energy structures in the respective clusters by affinity propagation, with the symbol size scaled by the accessible surface area. Here, the porosity spaces are defined by 4 descriptors: crystal density, pore diameter D_f, total surface area, and total pore volume.

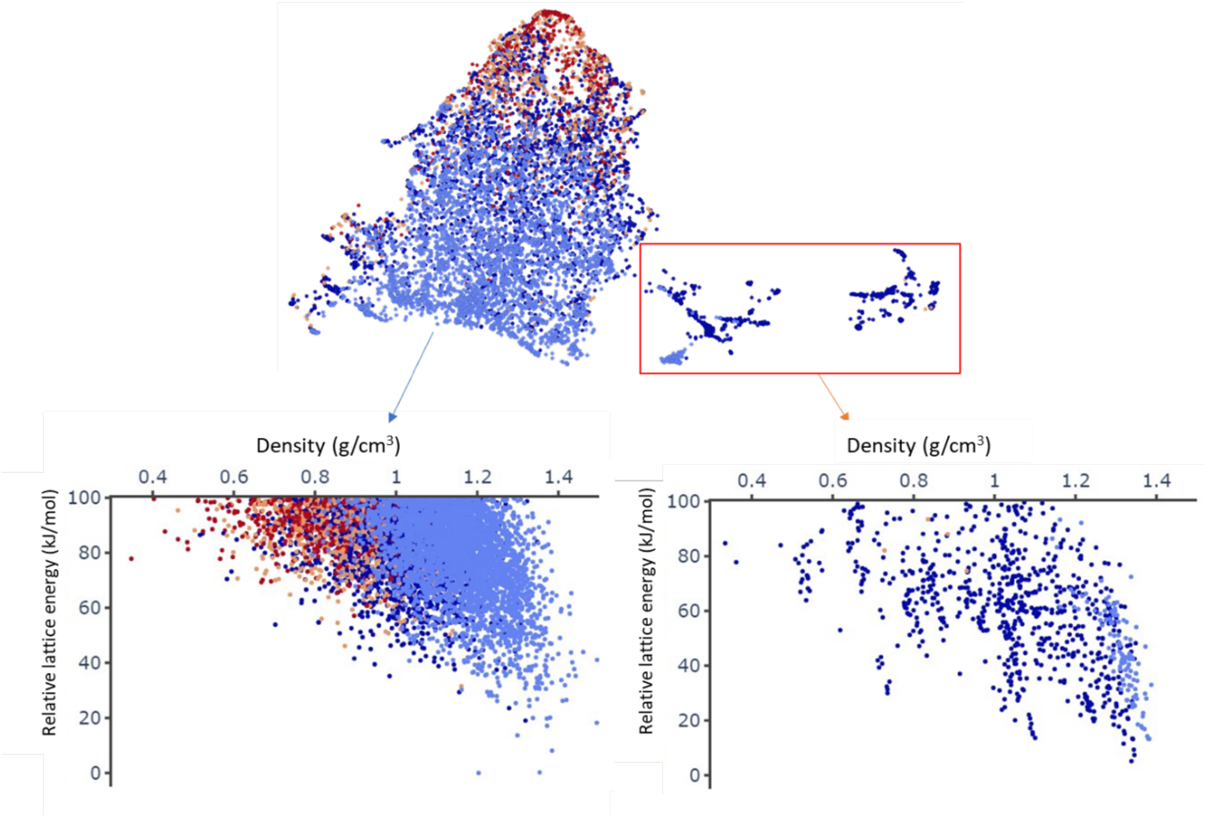

Supplementary Figure 14. Top: 2D UMAP embedding of the SOAP space of TH4, color-coded by the pore dimensionality (light blue, 0D; blue, 1D, orange, 2D; red, 3D). Bottom: energy–density landscapes correspond to the regions marked out in the top panel, color-coded by the pore dimensionality. Here, the cut-off radius used in calculation of the SOAP descriptors is 8.0 Å, while a radius of 6.0 Å was used for Figure 5 (main text), using the dscribe python package (https://singroup.github.io/dscribe).

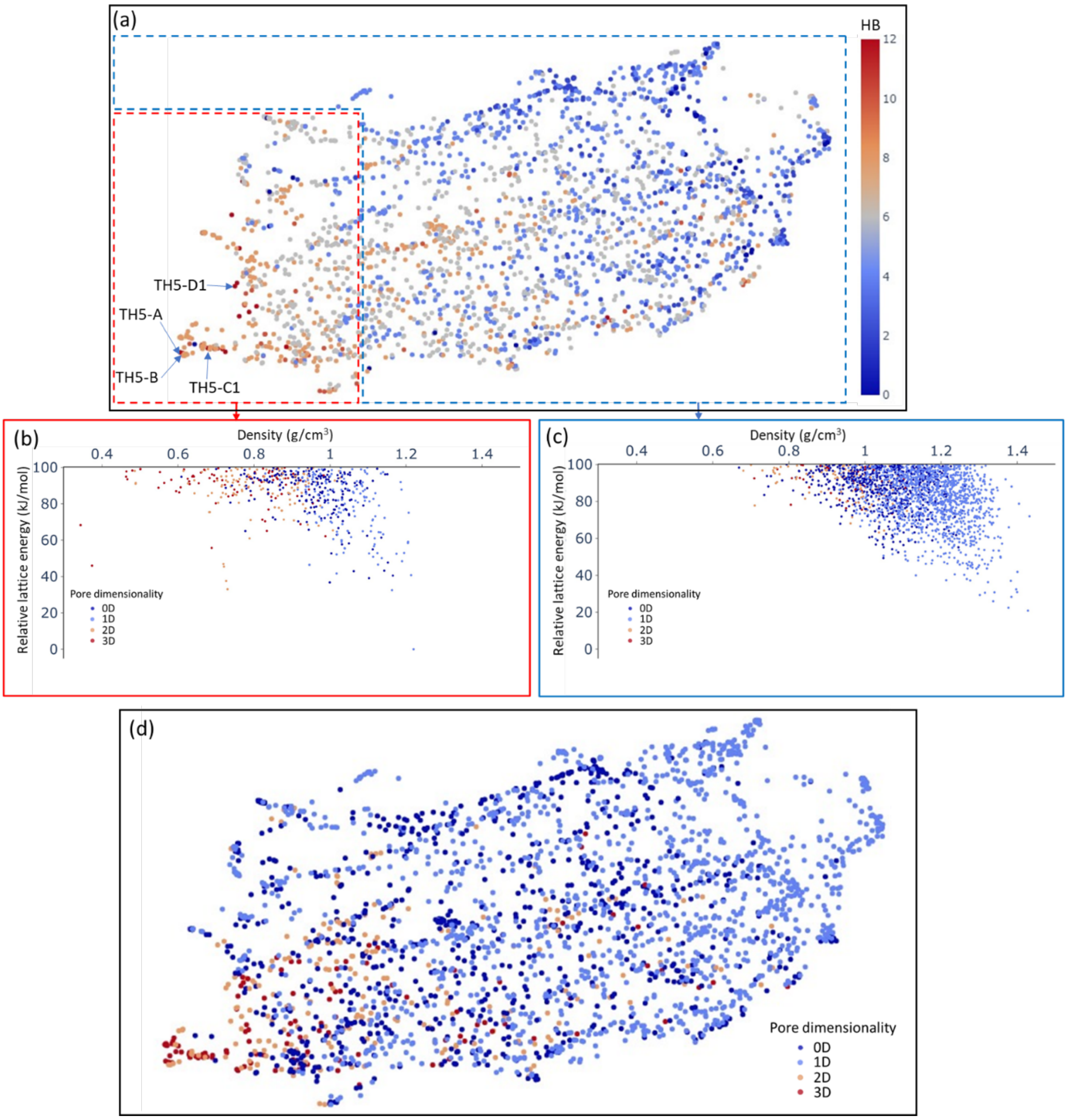

Supplementary Figure 15. (a) 2D UMAP embedding of the SOAP space of **TH5**, color-coded by the number of intermolecular hydrogen bonds (HB). (b), (c) Energy–density landscapes correspond to the regions marked out in (a), color-coded by the pore dimensionality. (d) 2D UMAP embedding, the same as the one in (a), of the SOAP space of **TH5**, color-coded by the pore dimensionality.

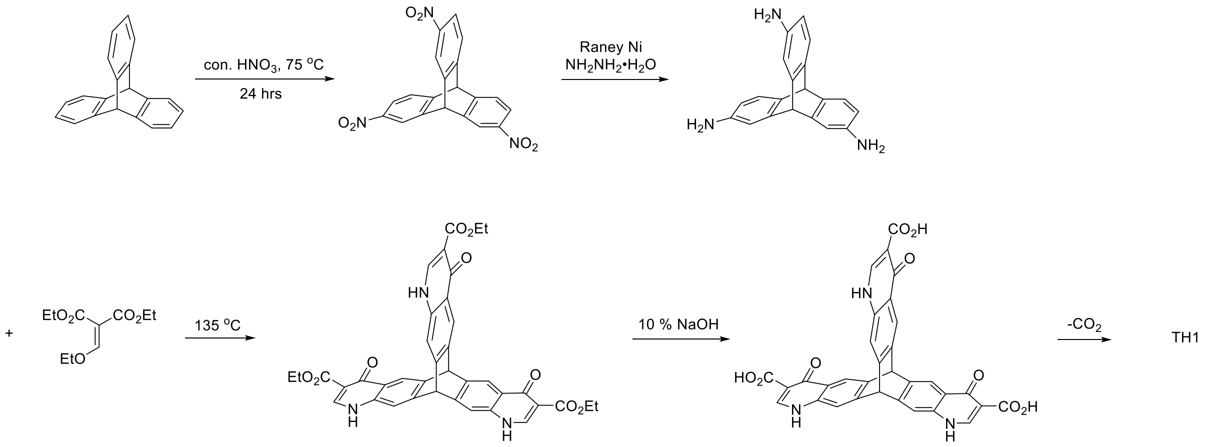

Supplementary Figure 16. Potential synthetic route to **TH1**.

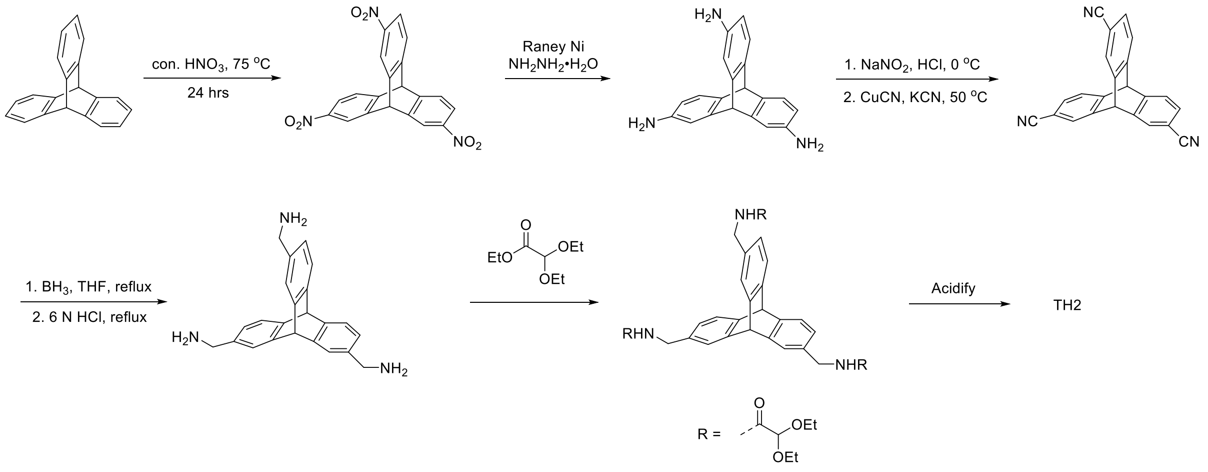

Supplementary Figure 17. Potential synthetic route to **TH2**.

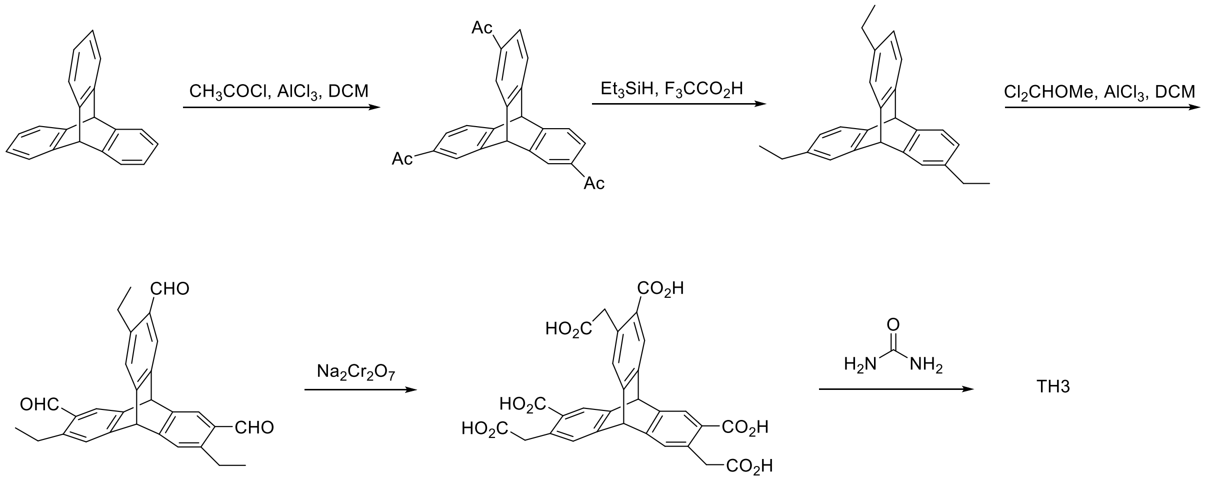

Supplementary Figure 18. Potential synthetic route to **TH3**.

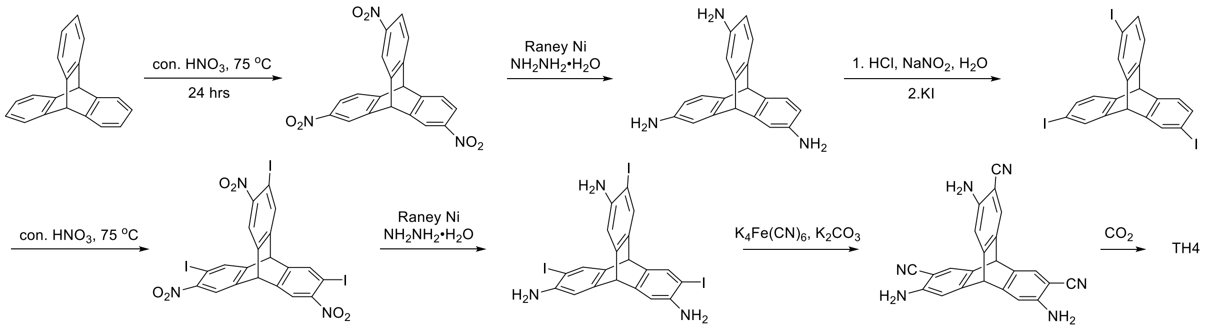

Supplementary Figure 19. Potential synthetic route to **TH4**.

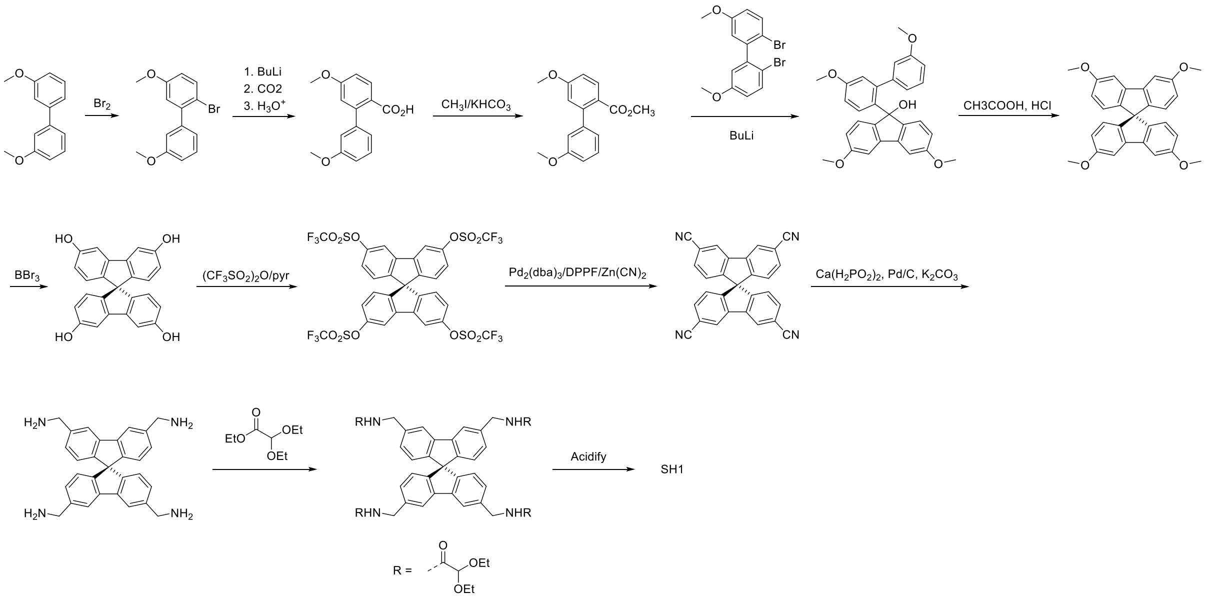

Supplementary Figure 20. Potential synthetic route to **SH1**.

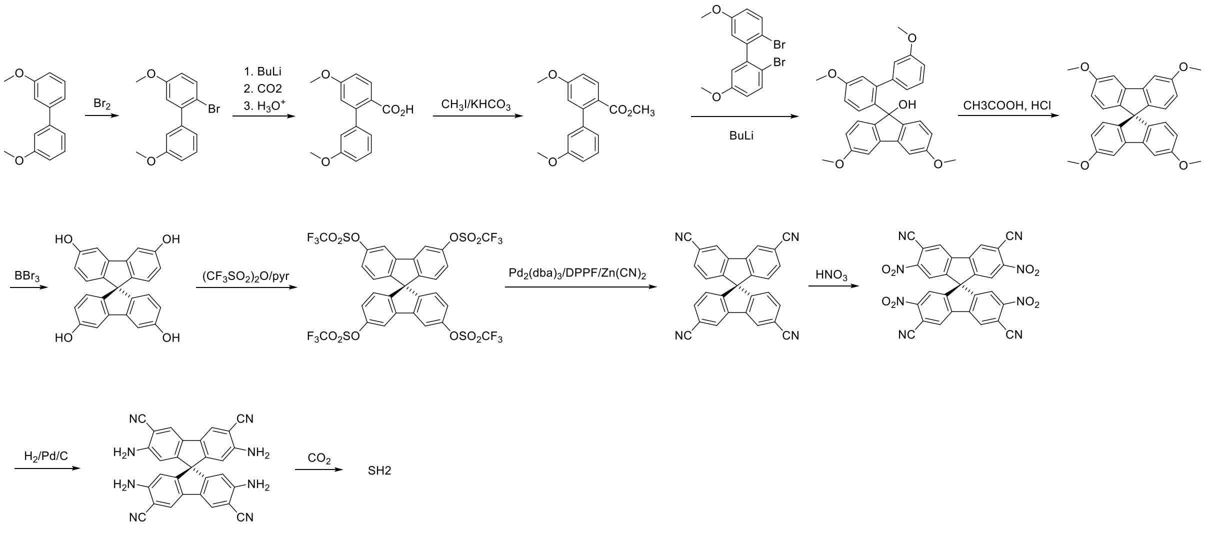

Supplementary Figure 21. Potential synthetic route to **SH2**.
